# HSG-ON: Hierarchical Scene Graph-Based Object Navigation

**DOI:** 10.3390/s26061755

**Published:** 2026-03-10

**Authors:** Seokjoon Kwon, Hee-Deok Jang, Dong Eui Chang

**Affiliations:** School of Electrical Engineering, Korea Advanced Institute of Science and Technology, Daejeon 34141, Republic of Korea; jun115533@kaist.ac.kr (S.K.); jhd6844@kaist.ac.kr (H.-D.J.)

**Keywords:** zero-shot object goal navigation, hierarchical scene graph, embodied ai, large vision language model, large language model, robotics

## Abstract

For a robot to operate effectively in human-centric environments, finding objects based on natural language is essential. Zero-shot object goal navigation is a significant challenge where robots must find unseen objects in new environments without prior knowledge. Existing methods often struggle with strategic exploration, leading to inefficient searches. In this study, we propose a hierarchical scene graph-based navigation system to address this challenge. Our core innovations are twofold: dynamically constructing a three-layer “room–workspace–object” hierarchical scene graph without manually pre-tuned parameters, and introducing a novel workspace-based searching strategy. By evaluating semantic relevance at the workspace level rather than the object level, the robot infers probable containers for a target, enabling focused, human-like exploration. Simulation results demonstrate that our system significantly outperforms existing state-of-the-art methods. Quantitatively, our approach improves the Success Rate (SR) by 26.8% (SR 0.4859) under distance-constrained settings and by 20.2% (SR 0.7360) under unconstrained settings, compared to the best baselines. These results validate that our framework offers a robust solution for zero-shot object goal navigation.

## 1. Introduction

Zero-Shot Object Goal Navigation (ZSON) [1,2] is a task in which an agent must search for a target object unseen during training in an unseen environment. In recent years, it has been actively studied as an essential capability for human-friendly robots operating in indoor settings. Because of the zero-shot feature of ZSON, it demands not only a strong understanding of the environment but also intelligent decision making to explore promising areas where the target object may be located.

While various approaches have been proposed to tackle ZSON, they often struggle with strategic exploration due to a lack of structured spatial reasoning. For instance, grid map-based methods project 3D visual information onto 2D planes, which can dilute fine-grained semantic relationships. Conversely, object-based methods navigate based on isolated objects, often leading to inefficient and myopic search trajectories. Both paradigms overlook the way humans naturally search for objects. When a human searches for an “apple” in an unfamiliar room, they do not randomly scan every single object. Instead, they first identify potential container-like entities—such as a “dining table” or a “kitchen counter”—that are likely to hold the target object. Humans naturally rely on these intermediate level for reasoning to narrow down the search space.

The primary objective of this study is to bridge this gap by developing a navigation framework that intrinsically emulates human-like hierarchical reasoning. Although 3D scene graphs effectively represent physical environments for robotic agents [3,4,5], they typically flatten all objects into a single hierarchical level, lacking this crucial container-level abstraction. To leverage such human-like reasoning, we introduced the Hierarchical Scene Graph (HSG) structure in our prior work [6], which explicitly incorporates an intermediate workspace level between rooms and objects. The prior work demonstrated that the HSG’s structured spatial representation provides a powerful foundation for LLM-based task planning.

**Figure 1:**
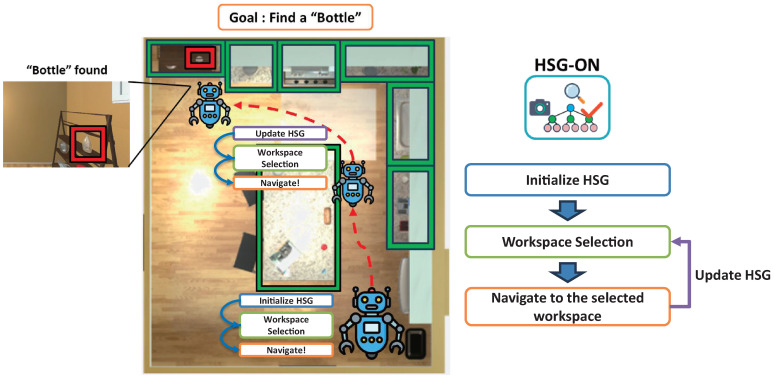
Overview of HSG-ON. The robot agent explores the environment to search for the target object by leveraging a hierarchical scene graph structured around workspace-level reasoning.

Building on this structure, we introduce a novel object navigation framework called HSG-ON (Hierarchical Scene Graph-based Object Navigation). Unlike our previous static representation, our method dynamically constructs and incrementally updates a 3D hierarchical scene graph using open-vocabulary segmentation models and LVLMs, resulting in a robust three-layer structure of room → workspace → object without requiring manually pre-tuned parameters. At each navigation step, the robot selects the most promising workspace node for exploration based on the current HSG and directs its attention accordingly. This approach, as illustrated in Figure 1, offers advantages over existing ZSON methods by enabling larger-scale movements while simultaneously considering all objects within each workspace. We validate our method through experiments in the AI2-THOR simulation environment [7] and verify that the system provides an effective solution for ZSON.

To explicitly highlight the innovation of our work compared to existing approaches, the main contributions of this paper are summarized as follows:

- Dynamic Hierarchical Scene Graph (HSG):Unlike existing static scene graphs, we propose a novel method to dynamically construct and incrementally update a three-layer (room–workspace–object) hierarchical scene graph using open-vocabulary segmentation and LVLMs, without relying on manually pre-tuned, environment-dependent parameters.
- Workspace-Level Reasoning for Navigation: We introduce HSG-ON, a zero-shot object goal navigation framework that emulates human-like search strategies by evaluating the semantic relevance at the workspace level, utilizing either semantic score-based or LLM-based selection methods.
- Empirical Validation of Efficiency: Through extensive experiments in the AI2-THOR simulation environment, we demonstrate that HSG-ON significantly outperforms state-of-the-art grid map-based and object-based baselines in both success rate and path efficiency.

The remainder of this article is organized as follows: Section 2 reviews related work in zero-shot object goal navigation and the application of scene graphs in robotics. Section 3 mathematically formulates the problem and introduces foundational concepts. Section 4 details the proposed HSG-ON framework, encompassing the dynamic construction of the hierarchical scene graph and the workspace selection strategies. Section 5 presents the experimental setup and discusses the evaluation results. Finally, Section 6 concludes the paper and outlines directions for future work.

## 2. Related Work

### 2.1. Zero-Shot Object Navigation

Early research on ZSON focused mainly on end-to-end learning approaches [1,2]. However, these methods are typically based on large-scale data collection and manual labeling, which presents challenges for real-world deployment [8]. As a result, recent research has largely diverged into two major directions: grid map-based methods and object-based methods.

Grid map-based methods typically project 3D perceptual information from the robot’s surroundings onto a 2D top-down grid map upon which the agent selects its next exploration location. Yu et al. [9] proposed L3MVN, a modular framework that leverages large language models (LLMs) to infer frontier relevance via zero-shot or feed-forward paradigms, enabling efficient object search without extensive training. Yokoyama et al. [10] introduced VLFM, a zero-shot semantic navigation framework that combines frontier-based exploration with a vision-language semantic value map employing a vision language model (VLM), BLIP-2 [11]. Yuan et al. [8] proposed GAMap, a zero-shot object goal navigation framework that guides exploration using multi-scale geometric parts and affordance attributes of the target object, rather than relying solely on categorical semantics or object detection.

Object-based methods, on the other hand, determine the next exploration region based on semantically meaningful entities detected in the environment, such as rooms or objects. For example, if the robot detects milk “in its current view and the target is bread”, the method may guide the robot to explore near the “milk”, assuming the two are co-located. Dorbala et al. [12] proposed LGX, a language-guided zero-shot object navigation framework where an LLM recommends navigation targets based on objects detected from RGB images. Cai et al. [13] proposed PixNav, a zero-shot object navigation framework that enables RGB-based navigation by leveraging pixel-level goals, VLMs for scene understanding, and an LLM-guided planner for long-horizon exploration.

### 2.2. Scene Graphs in Robotics

To improve the environmental understanding for robotic agents, scene graphs [14] have been widely adopted in robotics. Early scene graphs were constructed on 2D images, where objects were represented as nodes and their spatial or semantic relationships as edges [14,15,16]. These 2D scene graphs proved useful in image-level reasoning tasks such as image retrieval and visual question answering [14]. As their potential became more widely recognized, scene graphs were extended to 3D representations and increasingly applied in robotics [3,4,5,17]. Furthermore, the advancement of large vision-language models (LVLMs) has enabled the construction of 3D scene graphs enriched with semantic details by generating captions for each detected object [5]. Since 3D scene graphs effectively represent physical environments, a recent study [17] attempted to solve object search tasks using pre-built scene graphs. However, while some studies have proposed room–object scene hierarchies (e.g., house → room → object) [3,4], all objects within a room still exist at the same hierarchical level. This flat structure limits reasoning about containable groupings of objects, highlighting the need for intermediate workspace-level representations to achieve more human-like search behaviors.

To provide a structured overview of the current landscape, we categorize and compare the key characteristics of recent ZSON paradigms in Table 1. As summarized, while existing grid-based and object-based methods primarily rely on flat or non-hierarchical representations, our HSG-ON framework uniquely integrates a 3D Hierarchical Scene Graph with workspace-level reasoning to guide the exploration process more effectively.

## 3. Problem Formulation and Background

This section mathematically formulates the Zero-Shot Object Goal Navigation task and introduces the basic concept of scene graphs used in our framework.

### 3.1. Zero-Shot Object Goal Navigation

In the Zero-Shot Object Goal Navigation (ZSON) task, an autonomous agent is required to locate an instance of a target object category that was not seen during training, within a novel environment. Each task episode is defined as a tuple $E_{i}=\left\{e_{i},c_{i},p_{i}\right\}$, where $e_{i}$ denotes the environment, $c_{i}$ represents the semantic label of the target object category (e.g., “microwave”), and $p_{i}$ is the agent’s initial pose. The agent operates using egocentric sensory inputs, including RGB and depth images, as well as its current pose. At each timestep *t*, the agent receives an observation $O_{t}=\left\{I_{t},D_{t},p_{t}\right\}$ and must select a navigation action, where $I_{t}$, $D_{t}$, and $p_{t}$ denote an RGB image, a depth image, and the agent’s pose at timestep *t*, respectively. An episode is considered successful if the agent triggers the STOP action within a predefined threshold distance from any instance of the target object, all within a fixed number of action steps. This setting challenges the agent’s ability to reason semantically about the likely location of the target object without prior exposure to the specific environment or object instance.

### 3.2. Scene Graph

A scene graph, in general, is a structured representation of a visual or physical scene that captures its semantic composition through objects, their attributes, and inter-object relationships. Formally, a general scene graph is defined as a tuple G=(V,E), where $V=\{v_{1},v_{2},\ldots ,v_{N}\}$ is the set of *N* nodes, where each node represents an object instance and E⊆V×R×V is the set of directed edges. Each node $v_{i}$ is represented as a pair $v_{i}=\left(c_{i},A_{i}\right)$, where $c_{i}\in C$ is the semantic category label (e.g., “chair”, “table”), and $A_{i}\subseteq A$ is the set of attributes (e.g., “red”, “wooden”) associated with $v_{i}$. E⊆V×R×V is the set of directed edges, where each edge $e_{ij}=\left(v_{i},r_{ij},v_{j}\right)$ denotes a relationship $r_{ij}\in R$ between a pair of object nodes $v_{i}$ and $v_{j}$. The sets C, A and R denote the predefined categories of objects, attributes, and relationships, respectively. This graph-based abstraction can be instantiated from structured environmental data (e.g., pre-built maps, 3D scans), or dynamically constructed from sensory input such as RGB-D images and point clouds. Scene graphs constructed in this manner support contextual understanding by explicitly modeling the connectivity and semantics among entities in the environment, which is especially beneficial in applications such as object search and robot navigation.

## 4. Methodology

Our approach is founded on the conceptual framework of the Hierarchical Scene Graph (HSG) introduced in our previous work [6]. However, it is crucial to emphasize that the previous work focused strictly on high-level task planningwithin a fully pre-scanned, static environment, which was not designed for embodied exploration. In contrast, the proposed HSG-ON framework tackles a fundamentally different and more challenging problem: Zero-Shot Object Goal Navigation (ZSON) under partial observability.

To achieve this, HSG-ON transforms the static data structure into a dynamic perception-action loop. The original implementation of HSG faced significant limitations for navigation: it was static, meaning the graph is constructed only once from an initial 360 degree scan and could not be updated with new observations gathered during navigation. Second, its construction process relied on environment-specific thresholds that had to be determined empirically.

In this paper, we overcome these limitations by proposing an enhanced HSG generation method, which enables the HSG to be incrementally updated without manual pre-tuning. Furthermore, we introduce a novel active exploration mechanism—workspace-level searching strategy—that evaluates semantic relevance to iteratively guide the robot through unexplored areas. Building upon the representation, we introduce HSG-ON (Hierarchical Scene Graph-based Object Navigation), a framework designed to solve the challenging ZSON task. By leveraging the structured, workspace-level reasoning of the HSG, HSG-ON enables a more strategic and human-like search strategy. As we will demonstrate, this approach significantly outperforms existing grid map-based and object-based methodologies, establishing a more efficient solution for object navigation in previously unseen environments.

### 4.1. Hierarchical Scene Graph

**Figure 2:**
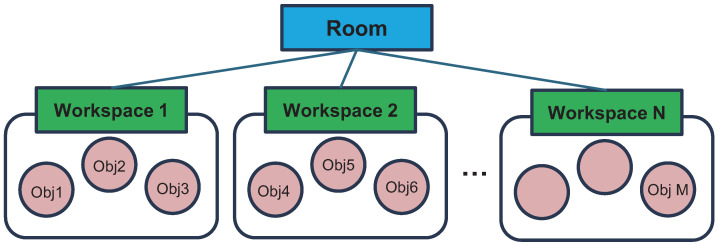
Illustration of HSG. Unlike a structure of a general scene graph, HSG has a hierarchical structure that includes a workspace node above the object nodes, serving as a potential container that contains objects.

Unlike conventional scene graphs that place all object instances on the same semantic level and connect them through pairwise spatial or semantic relationships [14,15], we define a HSG that explicitly introduces an intermediate abstraction level, “workspace”, between rooms and objects as illustrated in Figure 2. Formally, the hierarchical scene graph is defined as a tuple $G_{\mathrm{HSG}}=\left(V_{w},V_{o},E_{c}\right)$, where $V_{w}=\left\{w_{1},w_{2},\ldots ,w_{M}\right\}$ is the set of workspace nodes contained within the room; $V_{o}=\left\{o_{1},o_{2},\ldots ,o_{N}\right\}$ is the set of object nodes detected in the environment; and $E_{c}\subseteq V_{w}\times V_{o}$ is the set of edges encoding containment relationships connecting workspaces to the objects they contain. Each object node $o_{i}$ is associated with a category label $c_{i}\in C$ and a set of visual, spatial attributes $A_{i}\subseteq A$. Similarly, each workspace node $w_{j}$ is annotated with semantic labels and visual-spatial descriptors. While $E_{c}$ captures the hierarchical containment structure that guides navigation (e.g., “A mug is located in the table”), the graph can be readily extended to include inter-object edges $E_{r}\subseteq V_{o}\times R\times V_{o}$, which encodes spatial or functional relationships between objects, following standard definitions in prior works [5,15]. The HSG construction process consists of the following steps: nodes generation, local scene graph generation and global scene graph update.

#### 4.1.1. Nodes Generation

**Figure 3:**
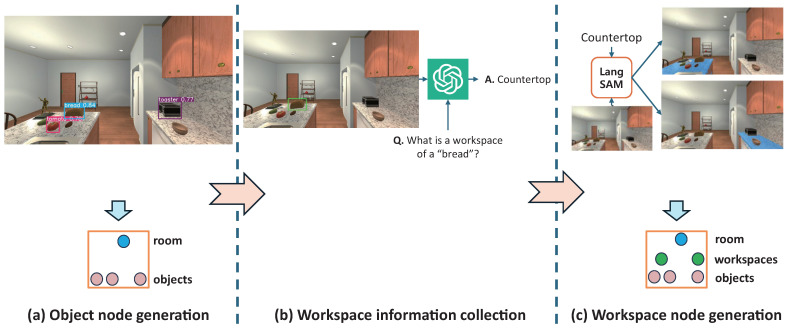
The detailed procedure of the node generation process. (**a**) Object node generation: The object detector identifies objects from the RGB image and extracts their visual and spatial features. (**b**) Workspace information collection: An LVLM infers the most probable workspace for each detected object using visual prompting. (**c**) Workspace node generation: The segmentation model extracts the precise pixel regions for the inferred workspaces, generating the final workspace nodes. The arrows indicate the sequential workflow of the process.

When a robot agent captures RGB-D data from its current position, it initiates the construction of a local HSG by generating object and workspace nodes, as illustrated in Figure 3. To generate object nodes, we employ the YOLOv8 [18] object detector trained to a sufficient extent on AI2THOR objects to identify objects within the input RGB image, creating a node for each detected instance. YOLOv8 was selected for its proven balance between accuracy and speed, serving as a reliable benchmark in embodied AI research. This choice ensures that our framework’s performance gains are primarily attributed to the proposed hierarchical reasoning rather than the specific detector, while providing a stable baseline for future sim-to-real transitions. Each object node includes the object name (class label) and encodes visual and spatial information. For the visual information, bounding box region corresponding to each detected object and a visual embedding vector extracted by a visual encoder of BLIP [19] are included. For the spatial features, the world frame points corresponding to the pixels within each bounding box and the object’s 3D position defined as the mean of the *x*, *y*, and *z* coordinates of the points are included. Additionally, a unique object ID (e.g., obj_0, obj_1) is assigned to each physically distinct instance. To maintain instance consistency for objects with the same name that appear across multiple images, we evaluate both visual similarity and spatial proximity, following the approach proposed in [5].

After the object nodes are generated, workspace information is inferred for each detected object. Specifically, the workspace associated with each object is estimated in textual form using a large vision-language model (LVLM), GPT-4o [20]. For each object detected in the RGB image, we iterate over all instances and query the LVLM using two inputs: (1) an image in which only the bounding box of each object is drawn, and (2) a textual prompt asking for the workspace of the object. The use of bounding-box-highlighted images serves as a form of visual prompting [21,22], which aims to enhance the model’s inference by directing its attention to a specific region of interest within the image. As illustrated in the center of Figure 3, if a piece of bread is detected in the RGB image, we draw a bounding box around the bread and present the modified image to the LVLM along with the question: “What is a workspace of the bread?”. The model then responds with a label such as “countertop” by recognizing the object and its surrounding contextual cues. This process is repeated for all detected objects, and the inferred workspace labels are recorded accordingly. After collecting workspace information for each object, we generate workspace nodes by segmenting the corresponding pixel regions in the RGB image. To achieve this, we employ the Language Segment Anything Model (LangSAM) [23], which integrates the Segment Anything Model 2 (SAM2) [24] with a grounding object detector to enable instance segmentation via textual queries. Specifically, for each unique workspace name obtained in the previous stage, we provide the workspace name as a text prompt along with the corresponding RGB image to LangSAM. The model then returns a binary mask of pixels and a bounding box corresponding to the queried workspace. Based on this output, we generate a workspace node, record its visual and spatial information in the same manner as object nodes, and assign it a unique workspace ID (e.g., ws_0, ws_1, …) to each physically distinct workspace.

#### 4.1.2. Local Scene Graph Generation

**Figure 4:**
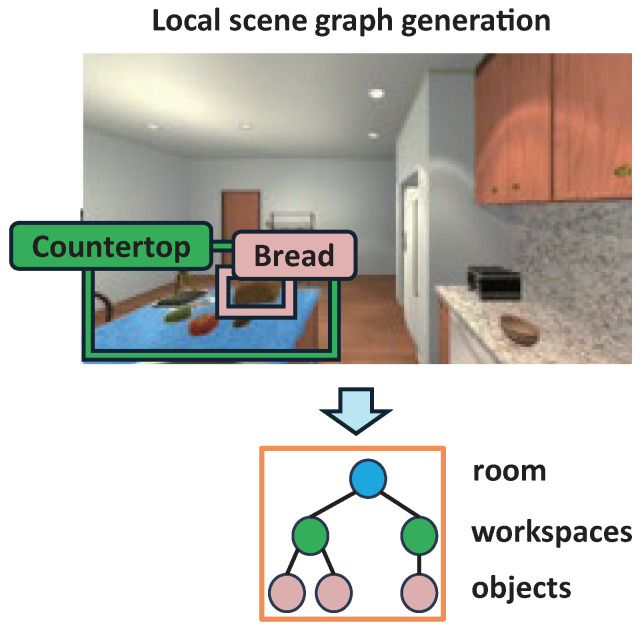
Illustration of local scene graph generation. The local scene graph is constructed by generating edges between objects and workspaces based on the spatial relationships of there bounding boxes.

After generating the object and workspace nodes, we construct the local scene graph by creating edges between each object and its corresponding workspace. For each object node, we first identify candidate workspace nodes whose names match the workspace label assigned to the object during the workspace inference stage, which is the second phase of the previous nodes generation process. To determine the appropriate workspace for each object, we compute the overlap between the object’s bounding box and the bounding boxes of the candidate workspace nodes. The workspace with the highest bounding box overlap is selected as the most likely container for the object. An edge is then established between the object node and the selected workspace node, adding the object as a child of the workspace in the scene graph. This process is repeated for all object nodes in the scene, resulting in the construction of a complete local scene graph composed of workspace–object relationships, as illustrated in Figure 4.

#### 4.1.3. Global Scene Graph Update

Once the local scene graph is constructed, it is integrated into the global scene graph. For each workspace node in the local graph, we check whether it corresponds to an existing workspace node in the global graph by comparing their positions in the world coordinate frame. If the distance between the two positions is below a predefined threshold (0.5 m), the two nodes are considered to represent the same physical workspace. This threshold was set to account for the general scale of workspaces, such as tables or countertops, found in common household environments. If a match is found, the object nodes associated with the local workspace are merged into the corresponding workspace node in the global graph. If no matching workspace exists, the local workspace node and its associated object nodes are added to the global graph as a new workspace subgraph. In this manner, the global scene graph is incrementally updated as the robot continues to explore the environment.

### 4.2. Hierarchical Scene Graph-Based Object Navigation

**Figure 5:**
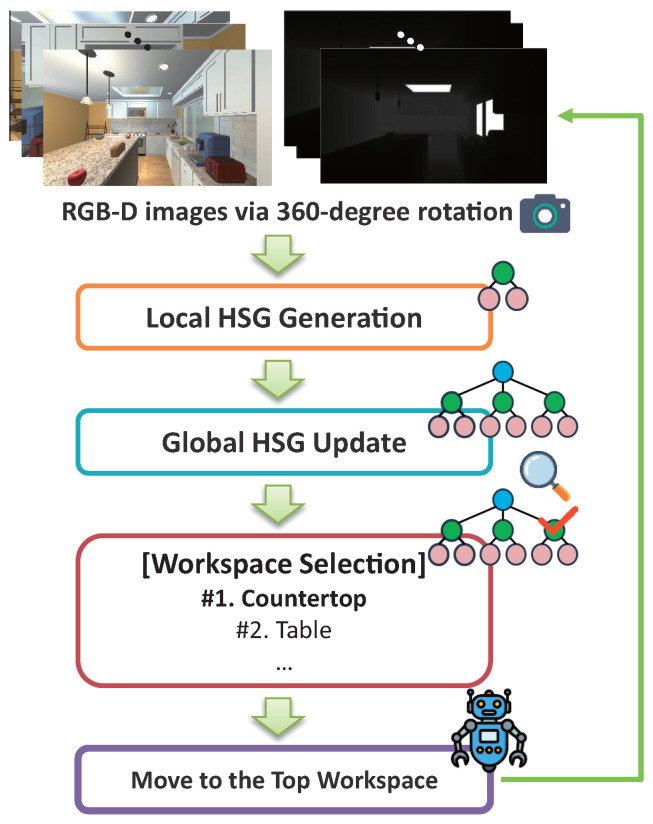
HSG-ON process. A local HSG is generated each time the robot agent observes RGB-D data and is subsequently merged into the global HSG. Based on the current global HSG, the most relevant workspace is selected, and the robot navigates toward it. This process repeats until the target object is found.

To guide the robot toward a specified target object, we introduce HSG-ON (Hierarchical Scene Graph-based Object Navigation). It first initializes a HSG by performing a 360-degree rotation in increments of $r^{\circ }$ at the initial position. Then HSG-ON identifies the workspace where the target object is most likely to be located, based on the initial HSG. The robot then navigates to the selected workspace and attempts to locate the target object. For path generation, the A* algorithm [25] is employed using the set of reachable points provided by AI2-THOR metadata. Upon arriving at the candidate workspace, the robot performs a 360-degree rotation in increments of $r^{\circ }$ in place to update the HSG with newly observed information. This multi-view rotational scanning specifically helps mitigate the issue of object occlusion by capturing the workspace from diverse viewing angles. However, as with human visual search, our framework is inherently limited to objects that are at least partially visible from the robot’s explored trajectories; objects that are completely enclosed or entirely hidden from all reachable viewpoints cannot be detected. Based on the updated HSG, the robot re-evaluates and selects the next most promising workspace, repeating this process until the target object is found. The process is illustrated in Figure 5. In our framework, we deliberately focus on selecting the single most probable workspace (i.e., top-1 selection) rather than a top-*k* sequence for two primary reasons. First, HSG-ON operates on a dynamic, real-time update mechanism. If the agent pre-computes and commits to a sequence of *k* workspaces, it fails to immediately leverage the newly discovered information acquired after visiting the first workspace. Re-evaluating the single best target at each step based on the most up-to-date global HSG ensures a highly reactive and rational exploration strategy. Second, unlike standard information retrieval tasks, an embodied physical robot is spatially constrained and can only navigate to one location at a time. Therefore, our framework is designed to commit to the single most promising target at each decision step. We introduce two approaches for selecting a workspace: semantic score-based selection and LLM-based selection.

#### 4.2.1. Approach 1. Semantic Score-Based Selection

To select the most appropriate workspace for navigating to the target object, we compute a workspace score for each workspace in the current HSG and select the one with the highest score. The workspace score, denoted by $WS_{i}$ for the *i*-th workspace, reflects the semantic relevance between the workspace (and its contained objects) and the target object described in natural language. It is defined as:

(1) $$WS_{i}=s_{w_{i}}+\frac{1}{N}\sum_{k=1}^{N}s_{obj_{k}},$$

where *N* is the number of objects contained in the *i*-th workspace; and the terms $s_{w_{i}}$ and $s_{obj_{k}}$ denote the semantic similarity scores for the *i*-th workspace and the *k*-th object respectively. More specifically, these scores are calculated as the cosine similarity between the text embedding vectors of their names and the target object’s name:

(2) $$\begin{array}{cc} s_{w_{i}} & =\frac{ws_{i}^{\mathrm{BLIP}}\cdot obj_{\mathrm{target}}^{\mathrm{BLIP}}}{∥ws_{i}^{\mathrm{BLIP}}∥\cdot ∥obj_{\mathrm{target}}^{\mathrm{BLIP}}∥}, \end{array}$$

(3) $$\begin{array}{cc} s_{obj_{k}} & =\frac{obj_{k}^{\mathrm{BLIP}}\cdot obj_{\mathrm{target}}^{\mathrm{BLIP}}}{∥obj_{k}^{\mathrm{BLIP}}∥\cdot ∥obj_{\mathrm{target}}^{\mathrm{BLIP}}∥}, \end{array}$$

where $ws_{i}^{\mathrm{BLIP}}\in R^{768}$, $obj_{k}^{\mathrm{BLIP}}\in R^{768}$, and $obj_{\mathrm{target}}^{\mathrm{BLIP}}\in R^{768}$ represent the text embedding vectors of the *i*-th workspace name, the *k*-th object name, and the target object name, respectively, extracted using the BLIP text encoder. Here, the dimension 768 is specifically selected as it corresponds to the default output embedding size of the pre-trained BLIP text encoder. After computing the workspace scores for all workspaces in the current global scene graph, the robot selects the workspace with the highest score for navigation. To avoid redundant exploration, previously visited workspaces are excluded from future selection. After moving to the selected workspace, the robot performs a 360-degree rotation in increments of $r^{\circ }$ to gather new observations and update the scene graph.

#### 4.2.2. Approach 2. LLM-Based Workspace Selection

**Figure 6:**
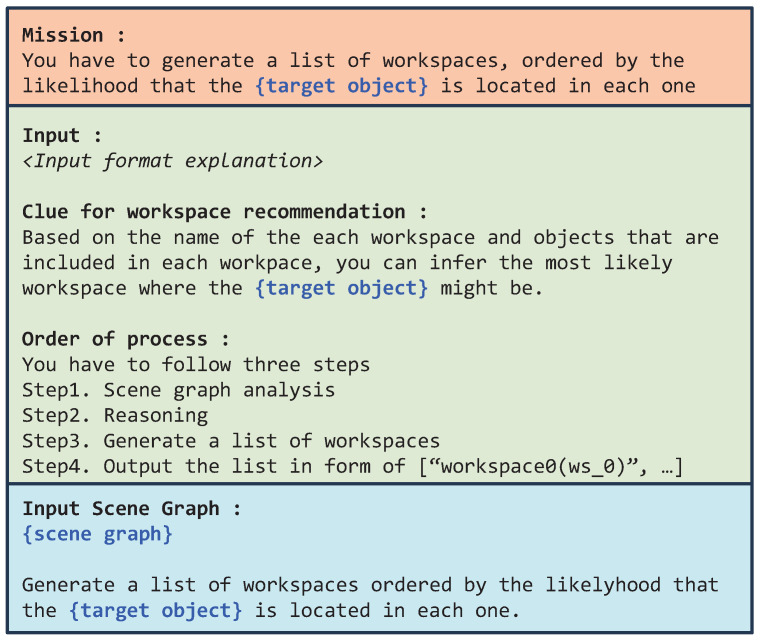
Prompt structure for LLM-based workspace selection. The prompt includes: (1) a mission description, (2) input format instructions, (3) reasoning cues for workspace selection, and (4) an ordered reasoning procedure. The target object name and the current HSG (in dictionary form) are inserted into the designated sections highlighted in blue.

In the second approach, we leverage a large language model (LLM) to select the most suitable workspace from the current HSG. The HSG is first converted into a Python dictionary of the form: $\left\{{\mathrm{key}}_{1}:{\mathrm{value}}_{1},{\mathrm{key}}_{2}:{\mathrm{value}}_{2},\cdots ,{\mathrm{key}}_{N}:{\mathrm{value}}_{N}\right\}$, where *N* is the total number of workspace nodes in the scene graph, ${\mathrm{key}}_{i}$ denotes the *i*-th workspace node, and ${\mathrm{value}}_{i}$ is the list of object nodes contained within that workspace. Each key takes the form workspace_name(workspace_id), and each list element is represented as object_name(object_id). The target object name and the HSG in dictionary form are then inserted into a prompt designed for the LLM to rank workspaces according to the likelihood of containing the target object. The structure of this prompt is illustrated in Figure 6. The “Input format explanation” in Figure 6 explicitly defines the data structure: it specifies the input as a Python dictionary in which each key represents a “workspace” (a localized area or container) and the corresponding values are lists of “objects” located within that workspace. Furthermore, it comments that all entities follow a structured name(id) format.

If the LLM returns a ranked list of workspace names in decreasing order of likelihood, the robot selects the top-ranked workspace as the next navigation target. If the selected workspace has already been visited, the robot moves to the next highest-ranked workspace in the list, continuing this process until an unexplored workspace is found. As in the first approach, once a workspace has been explored, it is excluded from future consideration.

## 5. Experiments

In this section, we evaluate the performance of the proposed HSG-ON framework. We first detail the experimental setup, including the simulation environment, target objects, baseline methods, and evaluation metrics. Subsequently, we present the comparative results under different navigation constraints and discuss the effectiveness of our hierarchical reasoning approach.

### 5.1. Experiment Setup

We evaluate our method using the iTHOR dataset from the AI2-THOR simulator [7]. We specifically selected iTHOR because it serves as a highly representative and widely adopted benchmark environment for embodied AI and object navigation tasks. Notably, it has been extensively utilized by foundational research in the Zero-Shot Object Goal Navigation (ZSON) domain, such as the pioneering work by Zhao et al. [2], as well as numerous other recent state-of-the-art studies [17,26,27,28,29]. Within this dataset, we focus on 60 rooms across two categories: bedrooms and kitchens. To construct the set of target objects for navigation, we first collect the names of all objects present in the 60 rooms using metadata provided by the simulator. Then we exclude object classes that appear in the training set of the object detector to construct the final set of target objects. This process enables evaluation under a zero-shot setting. The robot is allowed up to five movement steps to find the target object. In this context, a “movement” is explicitly defined as a single high-level navigation step: relocating from the robot’s current position to a newly selected target workspace. This counts as one movement step regardless of the low-level path length or the number of primitive actions required to reach the destination. An episode is considered successful if the robot approaches the target object within a distance of 1.2 m. Conversely, an episode is considered a failure if the robot does not reach the target within the five allowed steps. Across all rooms and target objects, a total of 178 episodes are conducted for each method.

We evaluate two object navigation approaches based on HSG: one using semantic scores, referred to as HSG-ON-semantic, and another leveraging a large language model (LLM), referred to as HSG-ON-LLM. We compare the methods with baselines based on grid map-based search as well as those based on object-based search. These specific baselines were carefully selected because they represent the recent state-of-the-art (SOTA) methodologies across the two dominant paradigms in ZSON. As baselines for grid map-based search, we implement VLFM [10] and GAMap [8]. The method of VLFM generates a semantic value map and a frontier map for object navigation. The value map encodes the likelihood of the target object’s presence on a 2D world grid based on vision-language embedding similarity. The frontier map identifies frontier points—midpoints of regions that lie between explored and unexplored areas. The robot selects the most promising frontier point for exploration based on these maps. GAMap gathers multiple semantic and geometric attributes of the target object using an LLM, then computes multi-scale relevance scores from vision-language embeddings across different image patches. These scores form a GAMap on a 2D world grid. The robot navigates to the location with the highest accumulated score, updating the map if the object is not found. For object-based search, we implement LGX [12] and PixNav [13] as baselines. In LGX, the robot performs a 360° rotation to detect surrounding objects. An LLM recommends the next object to approach based on the current observations. The robot navigates toward the suggested object and repeats the process until the target is found. In PixNav, a robot collects scene descriptions using an LVLM and queries an LLM to recommend an object or region to explore. A zero-shot object detector and SAM are then used to identify the center pixel of the suggested target. Then a pixel-level navigation model guides the robot toward the inferred goal location. In addition, we try a variant of HSG-ON-semantic that also falls under the object-based search category which we call non-hierarchical baseline. The variant computes the object score for each object based on nearby objects within a 0.7 m radius, without considering workspace hierarchy. This specific radius was empirically chosen as it reflects the average physical span of typical container-like furniture (e.g., dining tables, kitchen countertops) within the AI2-THOR environments, allowing the baseline to capture local object co-occurrences without explicitly modeling the container bounds. The object score for the *j*-th object, denoted $OS_{j}$, is defined as:

(4) $$OS_{j}=\frac{1}{M}\sum_{k=1}^{M}s_{obj_{k}},$$

where *M* is the number of objects within a 0.7 m radius of the *j*-th object including the *j*-th object itself, and $s_{obj_{k}}$ denotes the cosine similarity between the *k*-th object name and the target object name. This method focuses solely on local object-level context rather than workspace grouping.

For object detection, we use YOLO version 8. For both the LLM and LVLM components, we adopt GPT-4o. To ensure the reproducibility of the navigation results and to mitigate response sensitivity, the temperature parameter of the GPT4o was set to 0.0 for all experiments. The image and text encoders are based on the BLIP architecture. These models are consistently used across all evaluated methods, including HSG-ON-semantic and HSG-ON-LLM, to ensure a fair comparison with the baselines.

We adopt two standard evaluation metrics commonly used in the ZSON task [29,30]: Success Rate (SR) and Success weighted by inverse Path Length (SPL). SR measures the proportion of successful episodes, while SPL accounts for path efficiency by weighting each success by the ratio of the shortest possible path to the actual path taken. The metrics are formally defined as follows:

(5) $$\begin{array}{cc} \mathrm{SR} & =\frac{1}{E}\sum_{e=1}^{E}S_{e}, \end{array}$$

(6) $$\begin{array}{cc} \mathrm{SPL} & =\frac{1}{E}\sum_{e=1}^{E}\frac{S_{e}\cdot l_{e}}{\max(p_{e},l_{e})}, \end{array}$$

where *E* is the total number of episodes, $S_{e}\in \left\{0,1\right\}$ indicates whether episode *e* was successful, $l_{e}$ denotes the shortest path length from the start to the goal, and $p_{e}$ is the actual path length traversed by the robot in episode *e*.

We evaluate the proposed methods and comparison baselines under two experimental conditions: (1) distance-constrained navigation and (2) unconstrained navigation, as summarized in Table 2.

In the first setting, the robot is restricted to a maximum movement distance of 1.2 m per step. This distance is consistent with the success criterion and also matches the effective depth range used in the experiments. This constraint is essential for a fair comparison, as grid map-based methods inherently assume a depth-limited movement range per step. To ensure consistency, the proposed methods (HSG-ON-semantic, HSG-ON-LLM) and object-based baselines such as LGX, PixNav, and non-hierarchical baseline are modified to comply with this restriction. Specifically, these methods are adapted so that the robot stops once it reaches a point within 1.2 m from the initial position of each movement step, rather than directly navigating to the exact target position.

In the second setting, the robot is allowed to move without any distance limitations. This scenario assumes the use of high-performance perception hardware such as long-range depth cameras or LiDAR, which enables the robot to perceive and navigate over a much larger spatial range. In this setting, we compare HSG-ON-semantic, HSG-ON-LLM, and object-based methods (LGX, PixNav, non-hierarhical baseline) to evaluate their performance without being constrained by physical perception limits. This setting is designed to test the full potential of each method under ideal sensing conditions.

### 5.2. Results

Table 3 presents the performance comparison of the proposed and baseline methods under the two navigation settings: distance-constrained and unconstrained. Under the distance-constrained condition, where the robot is allowed to move up to 1.2 m per step, HSG-ON-LLM achieves the highest Success Rate (SR) of 0.4859 and Success weighted by inverse Path Length (SPL) of 0.4720, outperforming all baseline methods. The HSG-ON-semantic also demonstrates strong performance with an SR of 0.4326, surpassing both grid map-based and object-based baselines including VLFM (SR: 0.3833), GAMap (SR: 0.2090), LGX (SR: 0.3202), PixNav (SR: 0.3764), and non-hierarchical baseline (SR: 0.2697). In the unconstrained setting, which simulates scenarios with high-performance perception, both HSG-ON-LLM and HSG-ON-semantic exhibits notable performance gains. Concretely, HSG-ON-LLM achieves an SR of 0.7360 and an SPL of 0.4787, while HSG-ON-semantic follows closely with an SR of 0.6854 and SPL of 0.4433. These results confirm the effectiveness of our hierarchical reasoning strategy, especially when not limited by movement constraints.

The superior performance of our method can be attributed to its workspace-level reasoning using a hierarchical scene graph. By evaluating the semantic relevance of both workspace names and the objects they contain, the system is able to select exploration targets that are more likely to lead to successful object discovery. Unlike conventional object-based strategies that rely on sparse object-level signals, HSG-ON integrates spatial context and semantic relationships, enabling more intelligent and efficient navigation decisions. Furthermore, the hierarchical structure allows the robot to exploit broader spatial cues, such as container-like workspaces (e.g., tables, countertops), increasing the likelihood of discovering the target object even when it is not directly observable. This capability for semantic generalization is particularly advantageous in zero-shot settings, where neither the environment nor the target object category has been encountered during training. Collectively, these findings demonstrate that HSG-ON not only outperforms existing baselines across different experimental setups, but also provides a robust and efficient framework for ZSON task.

#### 5.2.1. Dataset Balance and Category-Wise Analysis

To further analyze the robustness of our dataset and the method’s performance across different object types, we categorized the target objects into two groups based on their physical properties—such as ‘pickupable’ and ‘moveable’—provided by the AI2-THOR metadata. We defined these categories as ‘Small/Movable’ (e.g., apple, cup, knife, laptop) and ‘Large/Stationary’ (e.g., cabinet, desk, fridge, microwave). As shown in Table 4, our dataset is reasonably balanced with a roughly 6:4 ratio (60.7% to 39.3%), which closely reflects the natural distribution of objects in real-world indoor environments where smaller, manipulable objects are more diverse than large furniture or appliances.

Furthermore, we analyzed the Success Rates across these categories as summarized in Table 5. Under the distance-constrained setting, the success rates for large/stationary objects are consistently higher across both methods (+4.7%p for HSG-ON-LLM and +3.6%p for HSG-ON-semantic). This is likely because larger objects are easier to observe from a distance and less prone to occlusion when the agent’s movement is restricted. However, under the unconstrained setting, the performance gap between the two categories diminishes significantly (within 1–2%p). This demonstrates that when allowed to fully explore and dynamically update the scene graph, HSG-ON effectively handles diverse object types, regardless of their size or mobility, without exhibiting significant bias.

#### 5.2.2. Ablation Study: The Role of Hierarchy

To explicitly evaluate the core contribution of our hierarchical structure, the non-hierarchical baseline serves as an ablation model for HSG-ON-semantic. By removing the workspace-level grouping, this variant relies solely on local object co-occurrences within a predefined radius. As shown in Table 3, eliminating the hierarchical reasoning results in a substantial performance degradation. Under the distance-constrained setting, the Success Rate drops significantly from 0.4326 (HSG-ON-semantic) to 0.2697 (non-hierarchical baseline), marking a 37.7% decrease, while the SPL drops from 0.2225 to 0.1364. A similarly sharp decline is observed in the unconstrained setting. This ablation study clearly demonstrates that explicitly modeling the container-like workspaces is the primary driving force behind the robust semantic reasoning and search efficiency of our framework.

#### 5.2.3. Computational Cost and Execution Time

To evaluate the practical feasibility of our framework, we analyzed the computational cost and wall-clock execution time. On average, a single episode takes 73.32 s to complete. During the scene graph construction phase, the system queries the LVLM (GPT-4o) an average of 16.3 times per episode, with an average response time of 3.584 s per query. For the decision-making step, HSG-ON-LLM invokes the LLM (GPT-4o) an average of 2.4 times per episode, with each query taking approximately 6.683 s.

While the overall processing time may seem intensive for strict real-time applications, it is important to note that most state-of-the-art ZSON frameworks similarly rely on heavy VLM/LLM queries. The core advantage of HSG-ON lies in its hierarchical reasoning, which significantly reduces the number of physical exploration steps required to find the target. In real-world robotics, physical locomotion and obstacle avoidance are typically more time-consuming and energy-intensive than computational inference. Therefore, minimizing the number of movement steps provides a substantial gain in overall task efficiency. Furthermore, to optimize processing time and API costs, our system intrinsically employs a caching strategy: once a workspace is evaluated and explored, it is explicitly excluded from future LLM queries, preventing redundant computations.

## 6. Conclusions

In this study, we have proposed HSG-ON (Hierarchical Scene Graph-based Object Navigation), a framework designed to address the task of ZSON. Our approach introduces a Hierarchical Scene Graph (HSG) representation that structures the environment into a layered graph of rooms, workspaces, and objects. The system incrementally constructs and updates a global HSG during navigation, and prioritizes promising workspaces for searching a target object—either through semantic score-based selection or LLM-based workspace selection. This structured reasoning mechanism allows the robot to emulate human-like search behavior by narrowing down the search space based on both object-level and workspace-level semantics. Experiments conducted in the AI2-THOR simulation environment demonstrate that HSG-ON outperforms existing baselines—including both grid map-based and object-based ZSON methods—in terms of success rate and task efficiency. Quantitatively, under the distance-constrained setting, our HSG-ON-LLM and HSG-ON-semantic approaches improve the Success Rate (SR) by 26.8% and 12.9%, respectively, compared to the best-performing baseline. Furthermore, in the unconstrained setting, they achieve SRs of 0.7360 and 0.6854, marking 20.2% and 11.9% improvements over the strongest baseline. In summary, by integrating a structured spatial representation with large-scale pretrained vision and language models, our framework provides a strategic and effective solution for intelligent object search in ZSON. Despite these advantages, the proposed method has certain limitations. First, as the system relies on line-of-sight observations, it inherently struggles to locate targets that are completely occluded, entirely enclosed within opaque containers, or too small to be detected by object detector. Second, the reliance on cloud-based LLMs/VLMs introduces network latency and API costs, averaging around 73 s per episode in our current setup. For future work, we plan to address these limitations by deploying the HSG-ON framework on a physical robot to evaluate its robustness against sensor noise. Furthermore, to enhance real-time performance and reduce operational costs, we will explore replacing the cloud-based foundation models with lightweight, on-device open-source LVLMs specifically fine-tuned for embodied navigation tasks.

## Figures and Tables

**Table 1:** Comparison of ZSON paradigms and representative methods with our proposed HSG-ON framework.

Paradigm	Representative Works	Scene Representation	Hierarchy	Targeting	VLM/LLM
End-to-End	Majumdar et al. [1], Zhao et al. [2]	Visual Features (Implicit)	×	Direct Action	×
Grid-based	VLFM [10], GAMap [8], L3MVN [9]	2D Frontier/Semantic Map	×	Frontier/Cells	✓
Object-based	LGX [12], PixNav [13]	Detected Object List/Pixel Goal	×	Object/Pixel	✓
Graph-based	HSG-ON (Ours)	3D Hierarchical Scene Graph	✓	Workspace	✓

**Table 2:** Summary of the experimental settings and their respective objectives.

Experimental Setting	Constraint	Objective
Distance-constrained	Max 1.2 m movement per step	Evaluate under realistic depth limits for fair comparison.
Unconstrained	No movement limits	Evaluate upper-bound performance assuming ideal perception.

**Table 3:** Comparison of performance under different conditions.

Metric	Condition	HSG-ON-LLM	HSG-ON-Semantic	VLFM	GAMap	LGX	PixNav	Non-Hierarchical Baseline
SR	Distance-constrained	0.4859	0.4326	0.3833	0.2090	0.3202	0.3764	0.2697
SPL	Distance-constrained	0.4720	0.2225	0.0930	0.1841	0.1829	0.2392	0.1364
SR	Unconstrained	0.7360	0.6854	–	–	0.5787	0.6124	0.4551
SPL	Unconstrained	0.4787	0.4433	–	–	0.3949	0.2221	0.2887

**Table 4:** Dataset balance: object category distribution.

Category	Example Objects	Proportion (%)
Small/Movable	Apple, Cup, Knife, Laptop, Potato	60.7%
Large/Stationary	Cabinet, Desk, Fridge, Microwave, Stool	39.3%

**Table 5:** Success rate by object category.

Constraint Setting	Method	Small/Movable	Large/Stationary	Gap (Large-Small)
Distance-Constrained	HSG-ON-semantic	42.1%	45.7%	+3.6%p
	HSG-ON-LLM	46.7%	51.4%	+4.7%p
Unconstrained	HSG-ON-semantic	69.4%	67.1%	−2.3%p
	HSG-ON-LLM	74.1%	72.9%	−1.2%p

## Data Availability

The data presented in this study are available on request from the corresponding author.

## Abbreviations

The following abbreviations are used in this manuscript:

ZSON	Zero-Shot Object Goal Navigation
HSG	Hierarchical Scene Graph
HSG-ON	Hierarchical Scene Graph-based Object Navigation
SR	Success Rate
SPL	Success weighted by inverse Path Length
